# Mechanisms of Metastasis in Colorectal Cancer and Metastatic Organotropism: Hematogenous versus Peritoneal Spread

**DOI:** 10.1155/2019/7407190

**Published:** 2019-09-19

**Authors:** E. Pretzsch, F. Bösch, J. Neumann, P. Ganschow, A. Bazhin, M. Guba, J. Werner, M. Angele

**Affiliations:** ^1^Department of General, Visceral, and Transplant Surgery, Ludwig-Maximilians-University Munich, Munich, Germany; ^2^Institute of Pathology, Ludwig-Maximilians-University Munich, Munich, Germany

## Abstract

Metastasis is the major cause of death in patients with colorectal carcinoma (CRC). The most common sites of metastasis are the liver and the peritoneum. Peritoneal carcinomatosis is often considered the end stage of the disease after the tumor has spread to the liver. However, almost half of CRC patients with peritoneal carcinomatosis do not present with liver metastasis. This brings up the question of whether peritoneal spread can still be considered as the end stage of a metastasized CRC or whether it should just be interpreted as a site of metastasis alternative to the liver. This review tries to discuss this question and summarize the current status of literature on potential characteristics in tumor biology in the primary tumor, i.e., factors (transcription factors and direct and indirect E-cadherin repressors) and pathways (WNT, TGF-*β*, and RAS) modulating EMT, regulation of EMT on a posttranscriptional and posttranslational level (miRNAs), and angiogenesis. In addition to tumor-specific characteristics, factors in the tumor microenvironment, immunological markers, ways of transport of tumor cells, and adhesion molecules appear to differ between hematogenous and peritoneal spread. Factors such as integrins and exosomal integrins, cancer stem cell phenotype, and miRNA expression appear to contribute in determining the metastatic route. We went through each step of the metastasis process comparing hematogenous to peritoneal spread. We identified differences with respect to organotropism, epithelial-mesenchymal transition, angiogenesis and inflammation, and tumor microenvironment which will be further elucidated in this review. A better understanding of the underlying mechanisms and contributing factors of metastasis development in CRC has huge relevance as it is the foundation to help find specific targets for treatment of CRC.

## 1. Introduction

Of all cancers, colorectal cancer (CRC) is the third most common with metastasis being the major cause of death in the majority of patients [1]. Common sites of distant metastasis are the liver and the peritoneum. About 20% of patients present with synchronous metastases, most commonly in the liver, and up to 60% of patients develop distant metastases within 5 years [2, 3]. Metastases in the peritoneum are found in 25% of patients with an inferior prognosis when compared to other metastatic sites [4]. However, although peritoneal carcinomatosis is considered to be an advanced stage of CRC, in 41–45% of metastasized CRC patients it is the only site of metastatic disease suggesting that peritoneal spread might be a locally advanced form of CRC without other distant metastases [5, 6]. These data point to the complexity of metastatic spread and many attempts have been made to understand the underlying principles of metastasis and organotropism. This review aims to give an overview of the current knowledge of the mechanisms of metastasis and metastatic organotropism in CRC.

## 2. Organotropism in Epithelial Tumors

Much progress has been made in understanding tumor biology and mechanisms of metastasis but knowledge on the factors influencing the metastatic route of tumor cells, especially in colorectal cancer, remains poor. Taken from recent findings on other epithelial tumors such as breast cancer and pancreatic cancer, metastatic organotropism is a nonrandom process regulated by different cancer cell intrinsic factors, the tumor microenvironment and the interaction between those cancer cell intrinsic factors and the tumor microenvironment [7]. Having only recently been discovered to play a pivotal role in the metastatic process, there are only very few studies on organotropism in malignant epithelial tumors so far. The next section tries to give an overview of the current knowledge on this hot topic and elaborate on mechanisms of organotropism that have been found to be of high relevance in malignant epithelial tumors.

### 2.1. Epithelial Characteristics

In pancreatic ductal adenocarcinoma (PDAC), metastatic organotropism to the liver and lung is dependent upon epithelial plasticity mediated by P120 Catenin (P120CTN). P120 Catenin is the binding and stabilizing partner of E-cadherin at the adherens junctions and has been described as a cancer candidate gene [8]. Mono-allelic P120CTN loss accelerates liver metastasis. However, loss of both P120CTN alleles results in the absence of liver metastasis. This might be due to the inability of cancer cells to establish new contacts with neighboring epithelial cells indicating that exhibition of epithelial characteristics is a premise to liver organotropism. Lung organotropism, however, seems to be independent of P120CTN expression and has also been demonstrated in cells with bi-allelic P120CTN loss [9]. The observation that liver metastasis is promoted by maintenance of an epithelial state through factors such as P120CTN and E-cadherin, while repression of E-cadherin is one of the main inductors of epithelial-mesenchymal transition (EMT), is a paradox and underscores the complexity of the metastatic cascade. To summarize, maintenance of epithelial characteristics seems to be a requirement for liver organotropism but negligible in lung organotropism.

### 2.2. Integrins

Lymphocyte function-associated antigen-1 (LFA-1) is an integrin with adhesive function that immune cells use to invade the liver under inflammatory conditions. Recently though, this integrin has been shown to be also expressed on solid tumors including CRC. A recent study conducted, using an in vitro assay and an in vivo mouse model, could show that decreased expression of the *β*2 subunit of LFA-1, which is required for integrin activation, adhesion, and signaling, correlates with a reduced activation of the liver endothelium and an improved local immune response in the liver. This results in a less tumor-promoting microenvironment. Furthermore, a reduction in early retention of cancer cells in the liver as well as a reduction in metastatic development and tumor size was observed [10]. In summary, the *β*2 subunit of LFA-1 integrin on colorectal tumor cells modulates liver organotropism and a decreased expression is associated with a reduction in liver metastasis.

In breast cancer, apart from cancer subtype and gene signature, metastatic organotropism was found to be regulated by molecular features such as chemokines and integrins. Binding of the chemokine CXCL12 to its receptor CXCR4 is associated with induction of liver metastasis and facilitates extravasation of tumor cells. *α*2*β*1 and *α*5*β*1 are integrin complexes being expressed on the cell surface where they interact with the liver stroma. Downregulation of these integrins results in reduced liver metastasis by preventing direct interactions of tumor cells with components of the extracellular matrix (ECM). *α*2*β*1 and *α*5*β*1 expression is upregulated by claudin-2, which is highly expressed in liver metastasis of breast cancer while expression in primary tumors is weak or absent [11].

### 2.3. Exosomal Integrins

Different exosomal integrin expression patterns have been linked to different routes of metastasis in breast cancer and pancreatic cancer, indicating that exosomal integrins could predict organ-specific metastasis underlying the phenomenon of organotropism [7]. Secreted from tumor cells, exosomal integrins initiate premetastatic niche formation which is defined as a tumor cell-free microenvironment in a putative organ of metastasis. By fusing with the target cells in this microenvironment, induction of inflammation and increment of vascular permeability through Src activation and S100 expression, exosomal integrins prime this microenvironment as a site for seeding of disseminated tumor cells [7]. Integrins *α*6*β*4 and *α*6*β*1, binding to lung-resident fibroblasts and epithelial cells, are associated with lung metastasis, and integrin *α*v*β*5, binding to Kupffer cells, is associated with liver metastasis [7].

### 2.4. Vascularisation of Metastasis

Breast cancer cells metastasizing to the liver show decreased mitochondrial metabolism and increased conversion of pyruvate into lactate. This altered metabolic program is due to expression of pyruvate dehydrogenase kinase-1 (PDK1), a target of HIF1a with VEGF and TWIST as downstream targets, enabling cancer cells to adapt to nutrient insufficiencies and hypoxia in the host stroma [11]. Interestingly, liver metastases emanating from breast cancer are not dependent on hypoxia and increased angiogenesis, while CRC liver metastases depend on these mechanisms [12]. In breast cancer, EGFR, COX2, and MMP-1 and MMP-2 have been shown to promote angiogenesis in lung metastasis. However, in order to overcome nonpermissive signals from resident cells of the lung, lung metastatic cancer cells express Coco and GALNTs [11]. To summarize, liver metastases in breast cancer show a nonangiogenic growth pattern independent of oxygen supply, whereas in CRC a high fraction of angiogenic liver metastases is found.

### 2.5. Cancer Stem Cell Phenotype

Metastatic spread in CRC has been shown to correlate with different expression of cancer stem cell markers. Liver metastasis in CRC is associated with the expression of cancer stem cell markers CD133, CD44, and *β*-catenin, whereas in peritoneal carcinomatosis no expression of these stem cell markers can be found. This might indicate that CRC with peritoneal carcinomatosis lack stem cell features needed for dissemination and, bearing the clinical course of the disease, could possibly be interpreted as an advanced stage of a locally aggressive tumor [13]. Multivariate analysis confirmed that a negative CD133 expression in the primary tumor of colorectal cancer patients with peritoneal carcinomatosis is an independent risk factor for reduced disease-free survival and can predict postoperative recurrence [14]. While the knowledge on stem cell markers associated with peritoneal carcinomatosis is still poor, a few other markers related to liver metastasis have recently been identified. Oct4 gene expression correlates with poor liver metastasis-free survival and Oct4-high cells actively form liver metastasis in vivo [15]. The stem cell markers Notch1 and ALDH1 correlate with lymph node metastasis, advanced stage, and tumor recurrence and represent an independent prognostic factor in colorectal carcinoma [16]. In summary, cancer stem cells seem to have a strong ability for migration and invasion and stem cell markers have been shown to be independent prognostic factors. However, the distribution of cancer cells to different metastatic sites as part of organotropism and the maintenance of biological features is also mediated by the microenvironment and part of a complex bidirectional mechanism [17].

## 3. Tumor Biology

**Figure 1:**
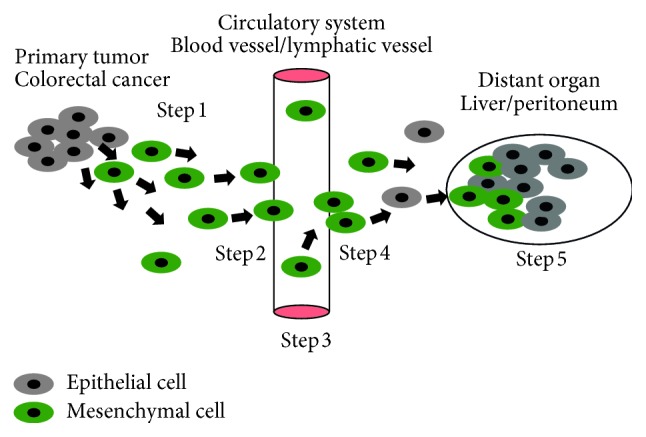
Invasion-metastasis cascade: local invasion of colorectal cancer cells into surrounding matrix (Step 1), intravasation into circulatory system (blood vessels/lymphatic vessels) (Step 2), systemic transportation (Step 3), extravasation (Step 4), colonization of distant organs (liver/peritoneum) (Step 5).

The process of tumor cells leaving their primary site and forming new colonies in distant tissues is described as invasion-metastasis cascade (Figure 1) [18]. This process consists of five steps: local invasion of tumor cells into surrounding matrix (Step 1), intravasation of tumor cells into circulatory system (Step 2), systemic transportation of tumor cells (Step 3), extravasation of tumor cells into parenchyma of distant tissue sites (Step 4), colonization of distant organs, and establishment of macroscopic tumors (Step 5) [19]. Although the underlying biology of some of these steps is yet to be defined, a few mechanisms have recently emerged and been increasingly recognized to play pivotal roles in the promotion of the invasion-metastasis cascade. However, the mechanisms determining hematogenous versus peritoneal spread are poorly understood.

### 3.1. Epithelial-Mesenchymal Transition

#### 3.1.1. Transcription Factors

**Figure 2:**
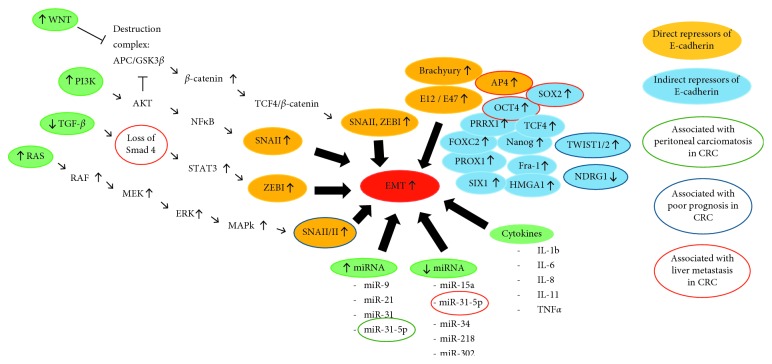
Regulation of EMT at different molecular levels: upregulation of the WNT pathway, PI3K/AKT pathway, and RAS/RAF/MEK/ERK/MAPK pathway and downregulation of the TGF-*β*/Smad pathway lead to the activation of EMT through downregulation of E-cadherin. E-cadherin repressors can be divided into direct and indirect repressors. EMT can also be regulated on a posttranscriptional and posttranslational level by miRNAs exerting activating and inhibiting functions. As part of tumor cell-tumor microenvironment interactions EMT can also be triggered by a variety of cytokines.

The epithelial-mesenchymal transition (EMT) is a key program that enables stationary epithelial cells to lose their cell-cell adherence and acquire mesenchymal properties that are essential for invasion and metastasis. These include enhanced mobility, invasiveness, increased resistance to apoptosis, and degradation and production of extracellular matrix (ECM) components [19]. The regulation of EMT occurs at different molecular levels (Figure 2).

E-cadherin, a transmembrane protein expressed by epithelial cells, plays an essential role in maintaining epithelial cell polarity and stabilizing cell-cell contacts allowing cells to sustain their epithelial state [20]. Downregulation of E-cadherin expression is associated with lymph node metastasis, advanced stage, poor differentiation, and vascular invasion [21]. A reduction in E-cadherin levels is commonly followed by an upregulation of N-cadherin, an adhesion molecule found in nonepithelial tissues. N-cadherin promotes tumor progression by enhancing fibroblast growth factor receptor (FGFR) signaling and nuclear *β*-catenin activity [20].

Transcription factors regulating E-cadherin expression in CRC can be divided into two groups: direct and indirect repressors. Direct repressors of E-cadherin bind directly to the E-cadherin promoter and include SNAIL (SNAI1,2), ZEB (ZEB1,2), E12/E47, Brachyury, and AP4 [22–29]. The indirect repressors TWIST (TWIST1,2), FOXC2, TCF4, SOX2, OCT4, Nanog, PROX1, SIX1, PRRX1, HMGA1, and Fra-1 have multiple specific targets and regulate the transcription of E-cadherin at different levels including the activation of direct repressors [30–39].

With respect to the clinical significance in CRC, the repressors AP4, SOX2, and OCT4 have been associated with liver metastasis [29, 32, 33]. Furthermore, an overexpression of the repressor TWIST1, that 85% of CRC patients show a moderate to strong expression of, is associated with nodal invasion, male sex, and poor outcome [40, 41]. Upregulation of SNAI2 significantly correlates with strong vimentin expression, and both SNAI2 and vimentin expression is associated with lymph node metastasis and poor prognosis [42].

Vimentin is an intermediate filament protein that is expressed in mesenchymal and neoplastic cells. Vimentin coexists and interacts with keratin-KRT14 through formation of an intracellular Vim/KRT14 hybrid and can be found in cells at the leading edge of migration. It is hypothesized that this hybrid formation disrupts the rigidity of keratin filaments, thereby promoting cellular migration. Expression of vimentin is regulated by the Vim gene regulating cancer cell migration and invasiveness. It has been shown that knockdown of the Vim gene disrupts keratinocyte colony growth and migration and represses EMT [43].

#### 3.1.2. Pathways

The EMT process is triggered by a multitude of extracellular signals in the tumor microenvironment with subsequent activation of all major cancer cell intrinsic signaling pathways. Considering the current status of literature it seems that all major signaling pathways have some implication in the EMT program of CRC and contribute differently to CRC progression. The following paragraph describes the signaling cascades that are currently considered to contribute significantly to the EMT program in a variety of epithelial tumors including CRC.

Aberrant activation of the canonical WNT pathway leads to the inhibition of the destruction complex that otherwise degrades *β*-catenin. Subsequently, free cytosolic *β*-catenin is translocated to the nucleus where it binds to the transcription factor TCF4 inducing WNT target gene transcription including activation of E-cadherin repressors ZEB1 and SNAI1 and upregulation of MT1-MMP9 and LAMC2 that are associated with CRC invasiveness [31, 44]. Inactivating mutations of tumor suppressor genes such as APC and AXIN2 lead to the upregulation of the canonical WNT pathway, thereby promoting EMT [44]. In summary, aberrant activation of the WNT pathway and *β*-catenin-dependent signaling promote tumor progression and are important EMT regulators in CRC [45].

Inactivation of the TGF-*β*/Smad signaling pathway leads to tumor progression, and mutations are found in 40–50% of CRC [46, 47]. In the absence of mutations, Smad2 and 3 are activated by TGF-*β* and transferred to the nucleus with Smad4 regulating transcription [48, 49]. In the presence of mutations, loss of Smad4 that usually suppresses STAT3 activation leads to aberrant activation of STAT3, which is linked to upregulation of ZEB1 expression, reduced E-cadherin, and enhanced N-cadherin and vimentin expression [50, 51]. With respect to clinical relevance in CRC, loss of Smad4 is found in 30% of metastatic CRCs and seems to be a predictor of liver metastasis [52]. Claudin-3 (CLDN3), an integral membrane protein and component of tight junctions, helps cells to remain in their epithelial state and functions as a suppressor of EMT. However, similar to Smad4, loss of CLDN3 expression leads to the induction of EMT through aberrant activation of STAT3. Its loss also leads to upregulation of the WNT pathway and predicts poor patient survival [53]. In conclusion, half of CRC patients present with mutations that lead to an inactivation of the TGF-*β*/Smad pathway which ultimately leads to the induction of EMT.

N-Myc downstream regulated gene 1 (NDRG1) has been shown to inhibit EMT, migration and invasion through attenuation of the above-mentioned pathways as well as the ErbB signaling pathway, and inhibition of prosurvival autophagic pathways in a variety of cancer cells [54–57]. In CRC patients, NDRG1 expression was found to be an independent prognostic factor for survival and tumor recurrence: CRC patients that are NDRG1 negative face a worse prognosis in cancer-free and overall survival [58, 59].

Another two major signaling pathways regulating EMT are the RAS/RAF/MEK/ERK/MAPK pathway and the PI3K/AKT pathway both triggered by growth factors such as EGF and FGF. Activation of the RAS/RAF/MEK/ERK/MAPK pathway leads to increased expression of SNAI1 and 2. The PI3K/AKT pathway can induce SNAI1 expression through NF-κB or through AKT inhibiting the destruction complex in the WNT pathway which ultimately leads to increased SNAI1 and ZEB1 expression [60, 61]. As described above, activation of SNAI1 and ZEB1 leads to repression of E-cadherin and induction of EMT in CRC. Also involved in these signaling networks, the fibroblast growth factor gene 18 (FGF18) shows elevated expression in CRC and acts as a downstream target of the WNT signaling pathway driving EMT [62].

Finally, we like to add some information on ADAM9 to this synopsis of pathways as this factor has recently emerged to play a significant role in a multitude of pathways regulating EMT and promoting metastasis in various cancers. ADAM9 is a membrane-anchored metalloprotease and part of the ECM compartment and has been shown to be upregulated in CRC promoting invasion [63, 64]. In CRC liver metastasis, ADAM9, secreted by hepatic stellate cells, binds to CRC cells and promotes carcinoma invasion through tumor-ECM interaction [65]. Studies on HCC cells and lung cancer cells showed that ADAM9, triggered by IL-6 which activates the JNK signaling pathway, induces the expression of the EMT-associated transcription factor SNAIL through NOX1 expression in the cell membrane and ROS production [66, 67]. ADAM9 promotes metastasis through enhanced CDCP1 expression, a promigratory transmembrane protein that is involved in cell-cell interaction and the regulation of anoikis resistance and is overexpressed in metastatic colon cancer [68].

#### 3.1.3. miRNAs

The EMT process can also be regulated on a posttranscriptional and posttranslational level by microRNAs (miRNAs) [19]. Overexpression of miRNA has-miR-31-5p in CRC with peritoneal metastasis inhibits EMT through suppression of c-MET, a kinase mediating EMT. In CRC with hepatic metastasis miRNA has-miR-31-5p is repressed supporting EMT possibly through upregulation of c-MET [69]. miR-200 is involved in the TGF-*β* pathway and targets ZEB1 as an inhibitor. Loss of tumor suppressor p53 leads to downregulation of miR-200 in CRC and increased expression of ZEB1, thereby promoting EMT [70–73]. Following loss of p53, other miRNAs are downregulated and support EMT in CRC. These include miR-34, miR-302, miR-15a, and miR-218. Downregulation of miR-34 leads to increased expression of SNAI1, cMET, and *β*-catenin [74]. Decreased miR-302 expression leads to upregulation of transcription factor AP4 and SNAI1 and increased expression of vimentin, all supporting EMT [75]. miR-15a targets AP4 thereby acting similarly to miR-302 [76]. miR-218 when downregulated no longer promotes apoptosis of cancer cells through c-FLIP [77]. miRNAs that when upregulated promote EMT including miR-21, miR-31, and miR-9. miR-21 with TGF-*β* as an upstream activator downregulates tumor suppressor Pdcd4 and consecutively promotes invasion, intravasation, and metastasis [78]. miR-31, also activated by TGF-*β*, targets SATB2, a gene that is linked to CRC metastasis [79]. miR-9 directly targets E-cadherin and inhibits its expression [35]. It should be noted that there are many more miRNAs involved in the regulation of EMT, but this review focused on the ones that have been most studied and established to play pivotal roles in the regulation of EMT. In addition, miRNAs have been shown to not only be involved in EMT but likely also in organotropism by targeting and altering the metabolism in the premetastatic niche and regulating cancer stem cell-mediated metastasis. Patients with CRC and peritoneal carcinomatosis show a high expression of miR-31-5p, whereas patients with CRC and liver metastasis only show a low expression of miR-31-5p. This suggests that miRNAs might contribute to defining the site of metastatic spread [69].

### 3.2. Angiogenesis

Angiogenesis is considered to be a crucial step in tumor growth and establishing a route of transport for metastatic tumor cells. Angiogenesis is not only necessary to sustain tumor growth but also to enable tumor cells enter the vasculature by forming new vessels that connect to the existent circulatory system. This vascular remodeling requires the activation of endothelial cells by proangiogenic factors [63].

The VEGF family members with VEGF A, B, C, D and PIGF are considered the most important inductors of angiogenesis. Other inductors include ANGPT and PLAT [80]. Hypoxia leads to the secretion of VEGF from hypoxic cells by mediation of HIF1a [81, 82]. Other triggers for VEGF expression are EGF, TGF-*α* and -*β*, PDGF, ILGF-1, and FGF [83]. Although there are still open questions on how all the VEGF family members are involved in angiogenesis, it has been shown that VEGF A binds to VEGFR2 on endothelial cells promoting migration, survival, and proliferation of those by inducing the MAPK and PI3K pathways. VEGF C acts through VEGFR3 promoting lymphangiogenesis [80].

There is increasing evidence that angiogenesis is not an isolated self-regulated process but a consequence of tumor-microenvironment interactions and is not limited to the generation of new vessels but can also accelerate metastasis.

A recent study highlighted the importance of tumor endothelial cells (TECs) that belong to the group of tumor stromal cells and are a result of tumor cell-microenvironment interactions. The function of TEC is not limited to the formation of new blood vessels to supply the tumor with nutrients and provide a route to disseminate but actually stimulate the tumor to metastasize. When tumor cells enter the circulatory system they physically touch TECs and interact with them through juxtacrine and paracrine signaling. TECs secrete biglycan, a small rich repeat proteoglycan, that stimulates tumor cells to metastasize through activation of NFκB and ERK signaling. Furthermore, TECs are able to downregulate tumor suppressive factors such as Slit2, thereby promoting tumor progression [84].

IL33 is another good example to illustrate the complexity and bidirectional interactions between tumor cells, microenvironment, and angiogenesis. This tumor-derived cytokine induces angiogenesis through recruiting myeloid cells that subsequently secrete VEGF. It also indirectly promotes liver metastasis in CRC by mobilizing macrophages and myeloid cells to remodel the stroma towards a pro-TME rather than changing the invasive or migratory properties and metastatic capabilities of the tumor cells [85]. Various tumor-associated leukocytes including macrophages, neutrophils, myeloid-derived suppressor cells and dendritic cells, mast cells, innate lymphoid cells, γ*δ*T cells, and natural killer cells have been identified to not only lead to an immunosuppressive TME but also contribute to angiogenesis by secretion of VEGF, chemokines, cytokines, proteases, structural proteins, and microvesicles [86].

Other factors that have already been mentioned in terms of their role in the EMT process but also contribute to angiogenesis in various cancers are ADAM9 and FGF18. In lung cancer metastasis, ADAM9 increases the expression of VEGF A, ANGPT2, and PLAT and activates EGFR [67]. FGF18 activates the ERK/MAPK pathway through binding to the FGFR3 receptor on endothelial cells thereby promoting angiogenesis in ovarian cancer. FGF18 can also activate NF-*κ*B leading to upregulation and secretion of proinflammatory cytokines with subsequent recruitment of tumor macrophages that secrete VEGF A and ECM degrading proteases like MMP9 to promote angiogenesis and creating a protumor microenvironment [87]. Another factor promoting angiogenesis through the ERK pathway is the calcium and integrin binding gene 1 (CIB1) that is upregulated in a variety of cancers, including CRC, often correlating with oncogenic KRas mutations [88]. On phosphorylation mediated by PKD2, CIB1 contributes to angiogenesis by mediating PKD2-induced VEGF production and secretion of tumor cells and VEGFR2 expression [89]. Homeodomain-interacting protein kinase-2 (HIPK2) has been demonstrated to function as a tumor suppressor in various types of cancer and its overexpression leads to the downregulation of VEGF promoter activity. Inhibition of HIPK2 by hypoxia results in induction of EMT and angiogenesis via WNT signaling and increased VEGF promoter activity [90]. A recent study could show that metastatic growth is in fact associated with hypovascularity. Vascularity decreases with increasing metastasis size. The bigger the lesion, the less it is surrounded by vessels, thereby resembling the primary tumor [91]. Although the underlying mechanism is still unknown it has been shown that in pancreatic cancer reduction of fibroblasts leads to increased vessel density pointing to fibroblasts exerting antiangiogenic effects [92].

A recent study could show that under hypoxic conditions CRC cells release exosomes into the tumor microenvironment that promotes angiogenesis. These exosomes, enriched with WNT4, lead to an induction of *β*-catenin signaling in endothelial cells and stimulate them to proliferate and migrate [93]. Considering the mechanisms of metastasis in CRC it seems that angiogenesis differs between hepatic and peritoneal spread, not in relevance though but in terms of time and order during the metastatic cascade. Liver metastases are considered the result of hematogenous dissemination. However, in order for tumor cells to be transported to the liver they first have to find access to the circulatory system. They also depend on the formation of new small vessels originating around the primary tumor that increase the likelihood of tumor cells entering the blood stream. Once they arrive at the liver, angiogenesis again is required in order for metastases to grow and proliferate [94]. The primary tumor itself can actively support this process by making the liver parenchyma more permissive for the homing and growth of metastasis by recruiting VEGFR-1 expressing haematopoietic progenitor cells that initiate the premetastatic niche [95, 96]. Patients with synchronous liver metastasis and the primary tumor still in situ show a significantly higher VEGFR1 and VEGF A expression in liver parenchyma adjacent to metastases than patients with metachronous liver metastases after resection of the primary tumor which underlines the tumor angiogenesis-promoting abilities of the primary tumor [97]. In contrast, peritoneal carcinomatosis is not considered a result of hematogenous dissemination but the consequence of lymphatic dissemination or tumor cell shedding into the peritoneal cavity with subsequent attachment to distant peritoneum. Hence, angiogenesis seems not to be a key element in the initial steps of the metastatic cascade. However, once the CRC cells arrived in the peritoneum via the mentioned mechanisms, they rely on angiogenesis to sustain proliferation and enable further growth [98].

## 4. Inflammation and Tumor Microenvironment

The interaction between cancer cells and their microenvironment is considered to be an essential component not only in the early steps of colorectal carcinogenesis but also in tumor progression and development of metastasis. This protumor microenvironment is composed of inflammatory and immune cells involving neutrophils and macrophages, carcinoma-associated fibroblasts (CAFs), environmental conditions such as hypoxia, soluble factors, signaling molecules, and ECM components [82]. Invasion of tumor cells requires the degradation of the basement membrane with subsequent migration of tumor cells through the stroma into neighboring tissues and with respect to liver metastases in CRC invasion into the vasculature requiring angiogenesis.

The process of stromal remodeling is regulated by TGF-*β* and PDGF, which is secreted by CAFs. Additionally, CAFs produce MMPs, MMP inhibitors, ECM components, cytokines, growth factors, and EMT-promoting factors facilitating not only cancer proliferation and invasion but also metastasis [99].

Through a complex network of soluble factors, macrophages are recruited into the tumor microenvironment (TME) where they exert different functions critically depending on the tumor stage. At early stages, the majority of tumor-associated macrophages (TAMs) are subtype 1 (M1) TAMs that possess proinflammatory characteristics and work to eliminate malignant cells; at later stages macrophages can switch to subtype 2 (M2) TAMS bearing immunosuppressive functions creating a microenvironment permissive of tumor growth. By secretion of ECM-degrading components (e.g., MMP1, 7, 9, 12) TAMs support cancer cells invade the stroma [100]. Furthermore, even proinflammatory M1 macrophages have been shown to promote EMT and activate the *β*-catenin/TCF4 pathway by releasing proinflammatory factors thereby supporting the metastatic cascade [101].

Besides their role in immune cell recruitment, cytokines can also act to induce EMT in malignant cells. IL-1b promotes EMT through ZEB1 activation, IL-11 via GP130/STAT3 signaling, and IL-6 via STAT3 activation and SNAI expression [102–104]. Downregulation of tumor suppressor p53 by IL-6 results in a reduction of E-cadherin and increased expression of SLUG, further promoting EMT [105]. TGF-*β*-induced SNAI expression can activate IL8 with subsequent activation of CXCR1 and induction of EMT through PI3K/AKT signaling [106, 107]. TNF-*α* can induce EMT directly by AKT/GSK-3b-mediated stabilization of SNAI and indirectly by increasing IL8 and CXCR1 [107, 108]. Loss of Smad 4 not only promotes EMT via the signaling pathways described above but also by increasing CCL15 expression leading to the recruitment of CCR(+) myeloid cells. These cells then help CRC cells to invade the stroma and metastasize to the liver by producing MMP9 [109].

In addition to the cancer cell-microenvironment interactions described above recent studies point to several more specific cells of the microenvironment that play distinct roles in the propagation of peritoneal metastasis. Among them are peritoneal macrophages (PMs), peritoneal mesothelial cells (PMCs), and peritoneal fibroblasts (PFBs). Peritoneal invasion is defined as tumor invasion beyond the peritoneal elastic lamina which creates the invasive tumor microenvironment facilitating tumor progression and metastasis. PMs form the first barrier to invasion by secretion of tumor necrosis factor (TNF). However, as described above, they can also switch to a tumor-promoting M2 phenotype driven by molecules released from CRC cells or by factors found in malignant ascites such as IL-6 and IL-10 [100]. Although the exact function of PMCs in the beginning stages of peritoneal carcinomatosis is not fully understood, PMCs are considered to play a major role in maintaining intraperitoneal homeostasis. Following PM activation, they secrete a variety of mediating molecules to the tumor microenvironment such as cytokines, chemokines, growth factors, ECM components, and adhesion molecules [100]. There is a debate however on PMCs exerting a protective role versus PMCs supporting cancer cell colonization. After tumor cell colonization, PFBs support tumor progression acting similar to CAFs. Recent studies suggest that CAFs might derive from PMCs; however, the mechanisms of this transformation are still unclear [100]. Focus on subpopulations of fibroblasts showed that subperitoneal fibroblasts (SPFs) play an active role in the process of tumor invasion. They show high gene expression of an ECM component and an upregulation of genes associated with cell contraction including *α*-SMA. Stroma-cell contractile ability and fibrosis with *α*-SMA expression promote cell migration, invasion, and accelerate metastasis [110]. CD90(+)/CD45(–) cells are a small subpopulation of mesothelilal-like cells (MLCs) found in the peritoneal fluid that also express characteristics of mesenchymal stem cells. In nude mice, intraperitoneal co-injection of MLCs and gastric cancer cells leads to enhanced tumorigenicity and an increased rate of metastatic formation in the peritoneal cavity. Following TGF-*β* stimulation, MLCs express collagen I, *α*-SMA, and vimentin and act similar to myofibroblasts. This way they seem to play a supportive role in the development of peritoneal metastasis by creating a permissive tumor microenvironment [111]. Lately focus has also been on peritoneal adipocytes (PAs) which have been found to promote proliferation and invasion of cancer cells through lipid internalization by gastric cancer cells thereby providing nutrients to the malignant compartment. The increased invasiveness is mediated by PI3K/Akt-signaling. Interestingly, only cells from the peritoneum of obese animals could promote tumor growth, underscoring the importance of host factors in the progression of malignant disease [112].

## 5. Development of Peritoneal Carcinomatosis: An Alternative Concept to the Invasion-Metastasis Cascade

As described above, hepatic spread is the result of the invasion-metastasis cascade, a concept that is applied to explain the mechanism of hematogenous dissemination in epithelial tumors including CRC. However, in the course of this article it becomes clear that in peritoneal carcinomatosis some of the steps simply differ from the proposed concept especially in terms of ways of detachment, dissemination, and attachment of tumor cells. The following paragraph introduces a concept specifically developed to explain the development of peritoneal carcinomatosis as opposed to the invasion-metastasis cascade. Also, differences between hematogenous and peritoneal spread in terms of transport and attachment of tumor cells to distant organ will further be elucidated below.

**Figure 3:**
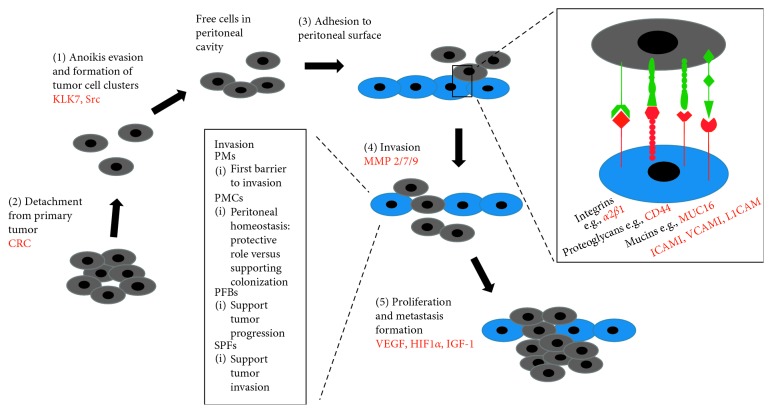
Development of peritoneal carcinomatosis: (1) detachment of colorectal cancer cells from their primary tumor and gain of motility, (2) anoikis evasion, (3) adherence to peritoneal surface, (4) invasion into peritoneum, and (5) proliferation and formation of peritoneal metastasis. Several adhesion molecules have been identified to be crucial in the adhesion process to the peritoneal surface, including integrins, proteoglycans, mucins, and members of the immunoglobulin superfamily. A multitude of cells and factors are involved in the process of invasion including peritoneal macrophages (PMs), peritoneal mesothelial cells (PMCs), peritoneal fibroblasts (PFBs), and subperitoneal fibroblasts (SPFs). Factors that have been identified to play a role in peritoneal carcinomatosis in CRC are highlighted red.

The development of peritoneal carcinomatosis involves five essential steps (Figure 3): (1) detachment of tumor cells from their primary tumor and gain of motility, (2) anoikis evasion, (3) adherence to peritoneal surface, (4) invasion into peritoneum, and (5) proliferation and formation of peritoneal metastasis [113]. The detachment of tumor cells from their primary tumor can be due to the following mechanisms: spontaneous exfoliation of tumor cells in CRC growing through the serosa (T4 stage), spontaneous tumor cell shedding into the lymphatics around the primary tumor as a result of increased interstitial fluid pressure, surgery-induced tumor spill due to opening of the tumor, transected lymphatics and blood vessels, or postoperative infections due to anastomotic leakage that have been shown to be associated with higher rates of tumor recurrence [98, 101, 113]. On a molecular level, the detachment of tumor cells and subsequent gain of motility is the result of a downregulation of cell-cell adhesion molecules (CAMs) as part of the EMT and changes in the cytoskeleton. Importantly, CAMs include integrins, cadherins, and selectins. Also involved in the EMT process are EGFR, c-MET, and especially in the context of peritoneal carcinomatosis, the EMT trigger TWIST [114].

## 6. Transport and Attachment of Tumor Cells to Distant Organ

In hematogenous spread platelets and neutrophils help tumor cells that have entered the circulatory system, so called circulating tumor cells (CTCs), avoid elimination by protecting them from shear stress or immune attacks from natural killer cells. TGF-*β* and platelet-derived growth factor (PDGF) released from platelets inhibit the activity of natural killer cells. By forming protective cloaks combined with fibrinogen, platelets also physically shield cancer cells from natural killer cells. Through TGF-*β*-induced activation of NF-κB in cancer cells the EMT program is reinforced and promoted so that CTCs will not lose their invasive characteristics needed for extravasation and metastatic colonization by returning to an epithelial state. Extravasation is further supported by ATP secretion from platelets increasing permeability of the vasculature and facilitating the entrance into the tissue [115]. Neutrophils have been shown to support metastatic spread by forming neutrophil extracellular traps that trap tumor cells in the blood stream, this way helping them adhere to endothelial cells, avoid natural killer cell attacks, and extravasate [115]. Due to poor adaptation to the new microenvironment most tumor cells are either eliminated after extravasation or enter a state of dormancy for years. These cells can be activated to grow by prometastatic changes in the microenvironment such as hypoxia, fibrosis, inflammation, and production of ECM components by CAFs which underlines the interdependence of cancer cells and their microenvironment as described above [115]. To evade anoikis in peritoneal carcinomatosis kallikrein-related peptidases (e.g., KLK7) are activated and tumor cells form clusters and continue to proliferate as opposed to the hematogenous dissemination where tumor cells are chemically and mechanically protected by platelets [114]. In addition, studies on CRC cell lines have shown that an upregulation of Src, a tyrosine kinase that plays a major role in cell-matrix and cell-cell contact-mediating adhesions, leads to an increased resistance to anoikis [116].

Recently, focus has been on identifying relevant adhesion molecules in peritoneal spread, and it has been shown that adhesion molecules differ in between hematogenous and peritoneal dissemination suggesting differences in attachment processes. In hematogenous spread, in CRC mainly to the liver, adherence to the endothelium in the hepatic sinusoids is required which involves CD44 binding to hyaluronan and the blood group antigens sLe^a^ and sLe^x^ binding to selectins and mucins binding to ECM components [113]. Important adhesion molecules in peritoneal carcinomatosis include integrins (e.g., *α*2*β*1) and integrin ligands, proteoglycans (e.g., CD44), members of the immunoglobulin superfamily (e.g., ICAM1, VCAM1, L1CAM), mucins (e.g., MUC16), and the epithelial cell adhesion molecule (EPCAM). There is an ongoing debate on the importance of each of these adhesion molecules with respect to the metastatic site. Data suggest that blood group antigens only play a role in hematogenous spread, whereas L1CAM and proteoglycans only contribute to peritoneal dissemination [113]. Although the exact differences remain unclear there is agreement on the importance of adhesion molecules in metastatic spread especially since free-floating tumor cells in the peritoneal cavity alone do not necessarily lead to peritoneal carcinomatosis [113]. The invasion into the peritoneum requires proteolytic enzymes such as matrix metalloproteases (e.g., MMP2/7/9) secreted by tumor cells or surrounding stromal cells. After invasion, tumor cells not only have to survive in the new environment but also sustain proliferation with IGF-1 and angiogenesis-promoting factors such as HIF1*α* and VEGF playing pivotal roles. IGF-1 mRNA has been shown to be overexpressed in peritoneal spread in comparison to liver metastases [114].

In search of novel biomarkers, DDR2, a type 1 collagen receptor tyrosine kinase associated with the Src pathway involved in a multitude of processes such as carcinogenesis and adhesion, was identified to be a driver gene of peritoneal carcinomatosis in gastric cancer. In CRC, high DDR2 expression was associated with higher frequencies of T4, lymph node metastasis, peritoneal spread, and poorer prognosis compared to low DDR2 expression, suggesting that DDR2 expression might be an effective therapeutic target [117].

## 7. Conclusion

**Figure 4:**
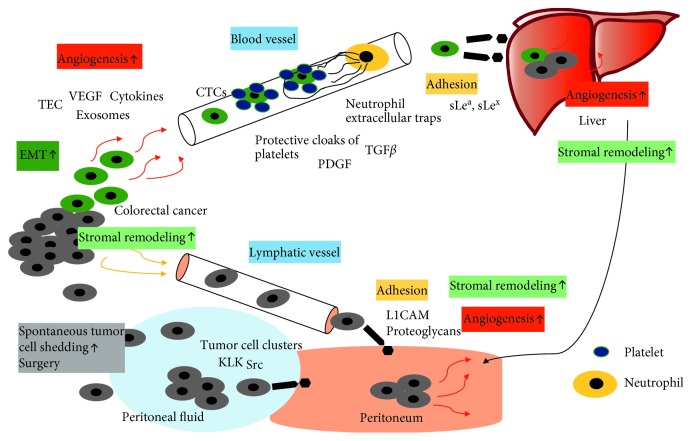
Mechanisms of metastasis in CRC—hematogenous versus peritoneal spread. Hematogenous spread: Tumor cells undergo EMT and leave the primary tumor. Through angiogenesis tumor cells find entrance into the circulatory system. During transport CTCs are protected by platelets and neutrophils from immune attacks and shear stress. Using distinct adhesion molecules CTCs then attach to the liver stroma and form colonies. Peritoneal spread: Tumor cells leave the primary tumor through spontaneous tumor cells shedding, surgical interventions, or exfoliation into the lymphatic system. In the peritoneal fluid, tumor cells form clusters to evade anoikis. Using certain adhesion molecules tumor cells attach to the peritoneum and use angiogenesis to further spread in the peritoneum. Factors that have been identified to play a role in specific parts of this multistep process in CRC are highlighted grey.

This review aimed to give an overview of the underlying principles of metastatic spread in CRC with respect to liver metastases versus peritoneal metastases (Figure 4). Current data suggest that EMT plays a major role in the beginning stages of tumor spread by enabling mobility and invasiveness. However, it is still unclear whether EMT equally contributes or differs in relevance to local tumor progression and peritoneal spread versus formation of distant metastasis, e.g., hematogenous spread.

Peritoneal carcinomatosis might be an advanced stage of local tumor progression and the result of spontaneous tumor cell shedding into the lymphatic system or exfoliation into the peritoneal fluid, whereas liver metastases are the result of hematogenous dissemination. During systemic transportation to the liver, CTCs are mechanically and chemically protected by platelets and neutrophils that help CTCs avoid shear stress and immune attacks of natural killer cells and support them in adherence to the endothelium and extravasation. During peritoneal spread, tumor cells form clusters to evade anoikis and continue to proliferate. Furthermore, recent studies suggest that adhesion molecules that help cells in the attachment process to their target organ differ significantly between hematogenous and peritoneal metastases. In liver metastases, the blood group antigens sLe^a^ and sLe^x^ might play an important role, whereas in peritoneal metastases L1CAM and proteoglycans could be a new focus. Angiogenesis is an important mechanism in CRC to sustain tumor growth. However, it seems that angiogenesis is also an essential step in the early steps of hematogenous metastasis formation by enabling tumor cells to connect to the preexistent vasculature by new vessel formation in the first place. Growing evidence points to the TME playing a crucial role in all stages of tumor development from tumor proliferation to metastasis formation to colonization of the peritoneum or distant organs. Tumor cells, immune cells, soluble factors, and ECM components seem to be all part of an ecosystem provided by the TME and described processes such as EMT or angiogenesis seem to be dependent on bidirectional interactions with the TME. Growing evidence suggests that peritoneal carcinomatosis in CRC is the terminal stage of a locally advanced tumor progress, whereas hepatic metastasis might be a hematogenously spreading CRC that could locally still be controlled. How CRC cells choose their route to disseminate and the underlying mechanisms of organotropism, especially in terms of CRC, have only very recently been started on uncovering. More experimental and clinical studies could contribute immensely to further understand and clarify the underlying principles of mechanisms of metastasis in colorectal cancer and metastatic organotropism. Better understanding of these mechanisms will help provide specific targets for therapeutic interventions in the future.

## References

[1] Ferlay J., Shin H.-R., Bray F., Forman D., Mathers C., Parkin D. M. (2010). Estimates of worldwide burden of cancer in 2008: GLOBOCAN 2008. *International Journal of Cancer*.

[2] Cook A. D., Single R., McCahill L. E. (2005). Surgical resection of primary tumors in patients who present with stage IV colorectal cancer: an analysis of surveillance, epidemiology, and end results data, 1988 to 2000. *Annals of Surgical Oncology*.

[3] Desch C. E., Benson A. B., Somerfield M. R. (2005). Colorectal cancer surveillance: 2005 update of an American society of clinical oncology practice guideline. *Journal of Clinical Oncology*.

[4] Segelman J., Granath F., Holm T., MacHado M., Mahteme H., Martling A. (2012). Incidence, prevalence and risk factors for peritoneal carcinomatosis from colorectal cancer. *British Journal of Surgery*.

[5] van Gestel Y. R. B. M., Thomassen I., Lemmens V. E. P. P. (2014). Metachronous peritoneal carcinomatosis after curative treatment of colorectal cancer. *European Journal of Surgical Oncology (EJSO)*.

[6] Thomassen I., Van Gestel Y. R., Lemmens V. E., De Hingh I. H. (2013). Incidence, prognosis, and treatment options for patients with synchronous peritoneal carcinomatosis and liver metastases from colorectal origin. *Diseases of the Colon and Rectum*.

[7] Hoshino A., Costa-Silva B., Shen T.-L. (2015). Tumour exosome integrins determine organotropic metastasis. *Nature*.

[8] Mann K. M., Ward J. M., Yew C. C. K. (2012). Sleeping Beauty mutagenesis reveals cooperating mutations and pathways in pancreatic adenocarcinoma. *Proceedings of the National Academy of Sciences*.

[9] Reichert M., Bakir B., Moreira L. (2018). Regulation of epithelial plasticity determines metastatic organotropism in pancreatic cancer. *Developmental Cell*.

[10] Benedicto A., Marquez J., Herrero A., Olaso E., Kolaczkowska E., Arteta B. (2017). Decreased expression of the *β*2 integrin on tumor cells is associated with a reduction in liver metastasis of colorectal cancer in mice. *BMC Cancer*.

[11] Chen W., Hoffmann A. D., Liu H., Liu X. (2018). Organotropism: new insights into molecular mechanisms of breast cancer metastasis. *NPJ Precision Oncology*.

[12] Stessels F., Van Den Eynden G., Van Der Auwera I. (2004). Breast adenocarcinoma liver metastases, in contrast to colorectal cancer liver metastases, display a non-angiogenic growth pattern that preserves the stroma and lacks hypoxia. *British Journal of Cancer*.

[13] Neumann J., Löhrs L., Albertsmeier M. (2015). Cancer stem cell markers are associated with distant hematogenous liver metastases but not with peritoneal carcinomatosis in colorectal cancer. *Cancer Investigation*.

[14] Nagata H., Ishihara S., Kishikawa J. (2018). CD133 expression predicts post-operative recurrence in patients with colon cancer with peritoneal metastasis. *International Journal of Oncology*.

[15] Fujino S., Miyoshi N. (2019). Oct4 gene expression in primary colorectal cancer promotes liver metastasis. *Stem Cells International*.

[16] Mohamed S. Y., Kaf R. M., Ahmed M. M., Elwan A., Ashour H. R., Ibrahim A. (2018). The prognostic value of cancer stem cell markers (Notch1, ALDH1, and CD44) in primary colorectal carcinoma. *Journal of Gastrointestinal Cancer*.

[17] Zhou Y., Xia L., Wang H. (2018). Cancer stem cells in progression of colorectal cancer. *Oncotarget*.

[18] Paschos K. A., Majeed A. W., Bird N. C. (2014). Natural history of hepatic metastases from colorectal cancer-pathobiological pathways with clinical significance. *World Journal of Gastroenterology*.

[19] Cao H., Xu E., Liu H., Wan L., Lai M. (2015). Epithelial-mesenchymal transition in colorectal cancer metastasis: a system review. *Pathology-Research and Practice*.

[20] Van Roy F. (2014). Beyond E-cadherin: roles of other cadherin superfamily members in cancer. *Nature Reviews Cancer*.

[21] Peña C., García J. M., Silva J. (2005). E-cadherin and vitamin D receptor regulation by SNAIL and ZEB1 in colon cancer: clinicopathological correlations. *Human Molecular Genetics*.

[22] Batlle E., Sancho E., Francí C. (2000). The transcription factor Snail is a repressor of E-cadherin gene expression in epithelial tumour cells. *Nature Cell Biology*.

[23] Cano A., Pérez-moreno M. A., Rodrigo I. (2000). The transcription factor Snail controls epithelial—mesenchymal transitions by repressing E-cadherin expression. *Nature Cell Biology*.

[24] Comijn J., Berx G., Vermassen P. (2001). The two-handed E box binding zinc finger protein SIP1 downregulates E-cadherin and induces invasion. *Molecular Cell*.

[25] Yang J., Mani S. A., Donaher J. L. (2004). Twist, a master regulator of morphogenesis, plays an essential role in tumor metastasis. *Cell*.

[26] Pe M. A., Locascio A., Rodrigo I. (2001). A new role for E12/E47 in the repression of E-cadherin expression and epithelial-mesenchymal transitions. *Journal of Biological Chemistry*.

[27] Wakeman J. A., Sarkar D., Shields B., Davies M. L. (2012). BRACHYURY confers cancer stem cell characteristics on colorectal cancer cells. *International Journal of Cancer*.

[28] Kilic N., Feldhaus S., Kilic E. (2011). Brachyury expression predicts poor prognosis at early stages of colorectal cancer. *European Journal of Cancer*.

[29] Jackstadt R., Röh S., Neumann J. (2013). AP4 is a mediator of epithelial-mesenchymal transition and metastasis in colorectal cancer. *The Journal of Experimental Medicine*.

[30] Watanabe T., Kobunai T., Yamamoto Y. (2011). Gene expression of mesenchyme forkhead 1 (FOXC2) significantly correlates with the degree of lymph node metastasis in colorectal cancer. *International Surgery*.

[31] Sanchez-Tillo E., de Barrios O., Siles L., Cuatrecasas M., Castells A., Postigo A. (2011). -catenin/TCF4 complex induces the epithelial-to-mesenchymal transition (EMT)-activator ZEB1 to regulate tumor invasiveness. *Proceedings of the National Academy of Sciences*.

[32] Han X., Fang X., Lou X. (2012). Silencing SOX2 induced mesenchymal-epithelial transition and its expression predicts liver and lymph node metastasis of CRC patients. *PLoS One*.

[33] Dai X., Ge J., Wang X., Qian X., Zhang C., Li X. (2013). OCT4 regulates epithelial-mesenchymal transition and its knockdown inhibits colorectal cancer cell migration and invasion. *Oncology Reports*.

[34] Meng H.-M., Zheng P., Wang X.-Y. (2010). Over-expression of Nanog predicts tumor progression and poor prognosis in colorectal cancer. *Cancer Biology and Therapy*.

[35] Lu M.-H., Huang C.-C., Pan M.-R., Chen H.-H., Hung W.-C. (2012). Prospero homeobox 1 promotes epithelial-mesenchymal transition in colon cancer cells by inhibiting e-cadherin via miR-9. *Clinical Cancer Research*.

[36] Ono H., Imoto I., Kozaki K. (2012). SIX1 promotes epithelial-mesenchymal transition in colorectal cancer through ZEB1 activation. *Oncogene*.

[37] Takahashi Y., Sawada G., Kurashige J. (2013). Paired related homoeobox 1, a new EMT inducer, is involved in metastasis and poor prognosis in colorectal cancer. *British Journal of Cancer*.

[38] Belton A., Gabrovsky A., Bae Y. K. (2012). HMGA1 induces intestinal polyposis in transgenic mice and drives tumor progression and stem cell properties in colon cancer cells. *PLoS One*.

[39] Diesch J., Sanij E., Gilan O. (2014). Widespread FRA1-dependent control of mesenchymal transdifferentiation programs in colorectal cancer cells. *PLoS One*.

[40] Go T., Bandre E., Cejas P. (2008). TWIST1 overexpression is associated with nodal invasion and male sex in primary colorectal cancer. *Annals of Surgical Oncology*.

[41] Okada T., Suehiro Y., Ueno K., Mitomori S., Kaneko S., Nishioka M. (2010). TWIST1 hypermethylation is observed frequently in colorectal tumors and its overexpression is associated with unfavorable outcomes in patients with colorectal cancer. *Genes, Chromosom Cancer*.

[42] Toiyama Y., Yasuda H., Saigusa S. (2013). Increased expression of slug and vimentin as novel predictive biomarkers for lymph node metastasis and poor prognosis in colorectal cancer. *Carcinogenesis*.

[43] Velez-Delvalle C., Marsch-Moreno M., Castro-Muñozledo F., Galván-Mendoza I. J., Kuri-Harcuch W. (2016). Epithelial cell migration requires the interaction between the vimentin and keratin intermediate filaments. *Scientific Reports*.

[44] Novellasdemunt L., Antas P., Li V. S. W. (2015). Targeting Wnt signaling in colorectal cancer. A review in the theme: cell signaling: proteins, pathways and mechanisms. *American Journal of Physiology-Cell Physiology*.

[45] Vincan E., Barker N. (2008). The upstream components of the Wnt signalling pathway in the dynamic EMT and MET associated with colorectal cancer progression. *Clinical and Experimental Metastasis*.

[46] Markowitz S., Wang J., Myeroff L. (1995). Inactivation of the type II TGF-beta receptor in colon cancer cells with microsatellite instability. *Science*.

[47] Markowitz S. D., Bertagnolli M. M. (2009). Molecular basis of colorectal cancer. *New England Journal of Medicine*.

[48] Hata A., Chen Y.-G. (2016). TGF-*β* signaling from receptors to smads. *Cold Spring Harbor Perspectives in Biology*.

[49] Brown K. A., Pietenpol J. A., Moses H. L. (2007). A tale of two proteins: differential roles and regulation of Smad2 and Smad3 in TGF-*β* signaling. *Journal of Cellular Biochemistry*.

[50] Zhao S., Venkatasubbarao K., Lazor J. W. (2008). Inhibition of STAT3Tyr705 phosphorylation by Smad4 suppresses transforming growth factor -mediated invasion and metastasis in pancreatic cancer cells. *Cancer Research*.

[51] Xiong H., Hong J., Du W. (2012). Roles of STAT3 and ZEB1 proteins in E-cadherin down-regulation and human colorectal cancer epithelial-mesenchymal transition. *Journal of Biological Chemistry*.

[52] Reinacher-schick A., Baldus S. E., Romdhana B. (2004). Loss of Smad4 correlates with loss of the invasion suppressor E-cadherin in advanced colorectal carcinomas. *The Journal of Pathology*.

[53] Ahmad R., Kumar B., Chen Z. (2017). Loss of claudin-3 expression induces IL6/gp130/Stat3 signaling to promote colon cancer malignancy by hyperactivating Wnt/*β*-catenin signaling. *Oncogene*.

[54] Mi L., Zhu F., Yang X. (2017). The metastatic suppressor NDRG1 inhibits EMT, migration and invasion through interaction and promotion of caveolin-1 ubiquitylation in human colorectal cancer cells. *Oncogene*.

[55] Jin R., Liu W., Menezes S. (2014). The metastasis suppressor NDRG1 modulates the phosphorylation and nuclear translocation of -catenin through mechanisms involving FRAT1 and PAK4. *Journal of Cell Science*.

[56] Kovacevic Z., Menezes S. V., Sahni S. (2016). The metastasis suppressor, N-MYC downstream-regulated gene-1 (NDRG1), down-regulates the ErbB family of receptors to inhibit downstream oncogenic signaling pathways. *Journal of Biological Chemistry*.

[57] Sahni S., Bae D.-H., Lane D. J. R. (2014). The metastasis suppressor, N-myc Downstream-regulated gene 1 (NDRG1), inhibits stress-induced autophagy in cancer cells. *Journal of Biological Chemistry*.

[58] Kovacevic Z., Richardson D. R. (2006). The metastasis suppressor, Ndrg-1: a new ally in the fight against cancer. *Carcinogenesis*.

[59] Mao Z., Sun J., Feng B. (2013). The metastasis suppressor, N-myc downregulated gene 1 (NDRG1), is a prognostic biomarker for human colorectal cancer. *PLoS One*.

[60] Suman S., Kurisetty V., Das T. P. (2014). Activation of AKT signaling promotes epithelial-mesenchymal transition and tumor growth in colorectal cancer cells. *Molecular Carcinogenesis*.

[61] Katoh M., Katoh M. (2006). Cross-talk of WNT and FGF signaling pathways at GSK3*ß* to regulate *ß*-catenin and SNAIL signaling cascades. *Cancer Biology and Therapy*.

[62] Shimokawa T., Furukawa Y., Sakai M., Li M., Miwa N., Lin Y. (2003). Advances in brief involvement of the FGF18 gene in colorectal carcinogenesis , as a novel downstream target of the ␤ -catenin/T-cell factor complex 1. *Cancer Research*.

[63] Weis S. M., Cheresh D. A. (2011). Tumor angiogenesis: molecular pathways and therapeutic targets. *Nature Medicine*.

[64] Li J., Ji Z., Qiao C., Qi Y., Shi W. (2013). Overexpression of ADAM9 promotes colon cancer cells invasion. *Journal of Investigative Surgery*.

[65] Mazzocca A., Coppari R., De Franco R. (2005). A secreted form of ADAM9 promotes carcinoma invasion through tumor-stromal interactions. *Cancer Research*.

[66] Dong Y., Wu Z., He M. (2018). ADAM9 mediates the interleukin-6-induced Epithelial-Mesenchymal transition and metastasis through ROS production in hepatoma cells. *Cancer Letters*.

[67] Lin C.-Y., Chen H.-J., Huang C.-C. (2014). ADAM9 promotes lung cancer metastases to brain by a plasminogen activator-based pathway. *Cancer Research*.

[68] Scherl-Mostageer M., Sommergruber W., Abseher R., Hauptmann R., Ambros P., Schweifer N. (2001). Identification of a novel gene, CDCP1, overexpressed in human colorectal cancer. *Oncogene*.

[69] Heublein S., Albertsmeier M., Pfeifer D. (2018). Association of differential miRNA expression with hepatic vs. peritoneal metastatic spread in colorectal cancer. *BMC Cancer*.

[70] Ling M., Liu C., Liang S., Kang X. (2012). miR-200c inhibits invasion and migration in human colon cancer cells SW480/620 by targeting ZEB1. *Clinical and Experimental Metastasis*.

[71] Gregory P. A., Bert A. G., Paterson E. L. (2008). The miR-200 family and miR-205 regulate epithelial to mesenchymal transition by targeting ZEB1 and SIP1. *Nature Cell Biology*.

[72] Chang C.-J., Chao C.-H., Xia W. (2011). p53 regulates epithelial-mesenchymal transition and stem cell properties through modulating miRNAs. *Nature Cell Biology*.

[73] Hur K., Toiyama Y., Takahashi M. (2014). MicroRNA-200c modulates epithelial-to-mesenchymal transition (EMT) in human colorectal cancer metastasis. *Gut*.

[74] Siemens H., Neumann J., Jackstadt R. (2013). Detection of miR-34a promoter methylation in combination with elevated expression of c-met and -catenin predicts distant metastasis of colon cancer. *Clinical Cancer Research*.

[75] Ma W., Liu B., Li J. (2018). MicroRNA-302c represses epithelial-mesenchymal transition and metastasis by targeting transcription factor AP-4 in colorectal cancer. *Biomedicine and Pharmacotherapy*.

[76] Shi L., Jackstadt R., Siemens H., Li H., Kirchner T., Hermeking H. (2014). P53-induced miR-15a/16-1 and AP4 form a double-negative feedback loop to regulate epithelial-mesenchymal transition and metastasis in colorectal cancer. *Cancer Research*.

[77] Meng Q., Chen Y., Lian B., Shang Y., Yang H. (2018). miR-218 promotes apoptosis of SW1417 human colon cancer cells by targeting c-FLIP. *Oncology Reports*.

[78] Asangani I. A., Rasheed S. A. K., Nikolova D. A. (2008). MicroRNA-21 (miR-21) post-transcriptionally downregulates tumor suppressor Pdcd4 and stimulates invasion, intravasation and metastasis in colorectal cancer. *Oncogene*.

[79] Yang M.-H., Yu J., Chen N. (2013). Elevated microRNA-31 expression regulates colorectal cancer progression by repressing its target gene SATB2. *PLoS One*.

[80] Dimova I., Popivanov G., Djonov V. (2014). Angiogenesis in cancer-general pathways and their therapeutic implications. *Journal of BUON*.

[81] Semenza G. L. (2002). Signal transduction to hypoxia-inducible factor 1. *Biochemical Pharmacology*.

[82] Mariani F., Sena P., Roncucci L. (2014). Inflammatory pathways in the early steps of colorectal cancer development. *World Journal of Gastroenterology*.

[83] Ferrara N., Davis-Smyth T. (1997). The biology of vascular endothelial growth factor. *Endocrine Reviews*.

[84] Maishi N., Hida K. (2017). Tumor endothelial cells accelerate tumor metastasis. *Cancer Science*.

[85] Peña M. M. O. (2017). IL-33 promotes growth and liver metastasis of colorectal cancer in mice by remodeling the tumor microenvironment and inducing angiogenesis. *Molecular Carcinogenesis*.

[86] Albini A., Bruno A., Noonan D. M., Mortara L. (2018). Contribution to tumor angiogenesis from innate immune cells within the tumor microenvironment: implications for immunotherapy. *Frontiers in Immunology*.

[87] Wei W., Mok S. C., Oliva E., Kim S.-h., Mohapatra G., Birrer M. J. (2013). FGF18 as a prognostic and therapeutic biomarker in ovarian cancer. *Journal of Clinical Investigation*.

[88] Zhu W., Gliddon B. L., Jarman K. E. (2017). CIB1 contributes to oncogenic signalling by Ras via modulating the subcellular localisation of sphingosine kinase 1. *Oncogene*.

[89] Armacki M., Joodi G., Nimmagadda S. C. (2014). A novel splice variant of calcium and integrin-binding protein 1 mediates protein kinase D2-stimulated tumour growth by regulating angiogenesis. *Oncogene*.

[90] Puca R., Nardinocchi L. (2008). Regulation of vascular endothelial growth factor expression by homeodomain-interacting protein kinase-2. *Journal of Experimental and Clinical Cancer Research*.

[91] Aiello N. M., Bajor D. L., Norgard R. J. (2016). Metastatic progression is associated with dynamic changes in the local microenvironment. *Nature Communications*.

[92] Rhim A. D., Oberstein P. E., Thomas D. H. (2014). Stromal elements act to restrain, rather than support, pancreatic ductal adenocarcinoma. *Cancer Cell*.

[93] Huang Z., Feng Y. (2017). Exosomes derived from hypoxic colorectal cancer cells promote angiogenesis through Wnt4-induced *β*-catenin signaling in endothelial cells. *Oncology Research Featuring Preclinical and Clinical Cancer Therapeutics*.

[94] Takeda A., Stoeltzing O., Ahmad S. A. (2002). Role of angiogenesis in the development and growth of liver metastasis. *Annals of Surgical Oncology*.

[95] Kaplan R. N., Riba R. D., Zacharoulis S. (2005). VEGFR1-positive haematopoietic bone marrow progenitors initiate the pre-metastatic niche. *Nature*.

[96] Kaplan R. N., Rafii S., Lyden D. (2006). Preparing the “soil”: the premetastatic niche. *Cancer Research*.

[97] Wal G. E. V. D., Gouw A. S. H., Moorlag H. E., Bulthuis M. L. C. (2012). Angiogenesis in synchronous and metachronous colorectal liver metastases: the liver as a permissive soil. *Annals of Surgery*.

[98] Lemoine L., Sugarbaker P., Van Der Speeten K. (2016). Pathophysiology of colorectal peritoneal carcinomatosis: role of the peritoneum. *World Journal of Gastroenterology*.

[99] George S. K., Theofilos P., Susan E. E., Richard K., Robert H. R., Eleftherios P. D. (2015). Cancer-associated fibroblasts drive the progression of metastasis through both paracrine and mechanical pressure on cancer tissue. *Molecular Cancer Research*.

[100] Mikuła-Pietrasik J., Uruski P., Tykarski A., Książek K. (2018). The peritoneal “soil” for a cancerous “seed”: a comprehensive review of the pathogenesis of intraperitoneal cancer metastases. *Cellular and Molecular Life Sciences*.

[101] Marcuello M., Mayol X., Felipe-Fumero E. (2018). Modulation of the colon cancer cell phenotype by pro-inflammatory macrophages: a preclinical model of surgery-associated inflammation and tumor recurrence. *PLoS One*.

[102] Li Y., Wang L., Pappan L., Galliher-Beckley A., Shi J. (2012). IL-1*β* promotes stemness and invasiveness of colon cancer cells through Zeb1 activation. *Molecular Cancer*.

[103] Calon A., Espinet E., Palomo-Ponce S. (2012). Dependency of colorectal cancer on a TGF-*β*-driven program in stromal cells for metastasis initiation. *Cancer Cell*.

[104] Rokavec M., Öner M. G., Li H. (2014). IL-6R/STAT3/miR-34a feedback loop promotes EMT-mediated colorectal cancer invasion and metastasis. *Journal of Clinical Investigation*.

[105] Brighenti E., Calabrese C., Liguori G. (2014). Interleukin 6 downregulates p53 expression and activity by stimulating ribosome biogenesis: a new pathway connecting inflammation to cancer. *Oncogene*.

[106] Cheng X.-S., Li Y.-F., Tan J. (2014). CCL20 and CXCL8 synergize to promote progression and poor survival outcome in patients with colorectal cancer by collaborative induction of the epithelial-mesenchymal transition. *Cancer Letters*.

[107] Bates R. C., DeLeo M. J., Mercurio A. M. (2004). The epithelial-mesenchymal transition of colon carcinoma involves expression of IL-8 and CXCR-1-mediated chemotaxis. *Experimental Cell Research*.

[108] Wang H., Wang H.-S., Zhou B.-H. (2013). Epithelial-mesenchymal transition (EMT) induced by TNF-*α* requires AKT/GSK-3*β*-Mediated stabilization of snail in colorectal cancer. *PLoS One*.

[109] Itatani Y., Kawada K., Fujishita T. (2013). Loss of SMAD4 from colorectal cancer cells promotes CCL15 expression to recruit CCR1+ myeloid cells and facilitate liver metastasis. *Gastroenterology*.

[110] Kojima M., Higuchi Y., Yokota M. (2014). Human subperitoneal fibroblast and cancer cell interaction creates microenvironment that enhances tumor progression and metastasis. *PLoS One*.

[111] Kitayama J., Emoto S., Yamaguchi H., Ishigami H., Watanabe T. (2014). CD90(+) mesothelial-like cells in peritoneal fluid promote peritoneal metastasis by forming a tumor permissive microenvironment. *PLoS One*.

[112] Zhang Y., Nowicka A., Solley T. N. (2015). Stromal cells derived from visceral and obese adipose tissue promote growth of ovarian cancers. *PLoS One*.

[113] Sluiter N., de Cuba E., Kwakman R., Kazemier G., Meijer G., te Velde E. A. (2016). Adhesion molecules in peritoneal dissemination: function, prognostic relevance and therapeutic options. *Clinical and Experimental Metastasis*.

[114] De Cuba E. M. V., Kwakman R., Van Egmond M. (2012). Understanding molecular mechanisms in peritoneal dissemination of colorectal cancer. *Virchows Archiv*.

[115] Lambert A. W., Pattabiraman D. R., Weinberg R. A. (2017). Emerging biological principles of metastasis. *Cell*.

[116] Sakamoto M., Takamura M., Ino Y., Miura A., Genda T., Hirohashi S. (2001). Involvement of c-src in carcinoma cell motility and metastasis. *Japanese Journal of Cancer Research*.

[117] Sasaki S., Ueda M., Iguchi T. (2017). DDR2 expression is associated with a high frequency of peritoneal dissemination and poor prognosis in colorectal cancer. *Anticancer Research*.

